# Phylogenomic Analysis Reveals Evolutionary Relationships of Tropical Drosophilidae: From *Drosophila* to *Scaptodrosophila*

**DOI:** 10.1002/ece3.71100

**Published:** 2025-03-10

**Authors:** Matsapume Detcharoen, Pairot Pramual, Areeruk Nilsai

**Affiliations:** ^1^ Division of Biological Science, Faculty of Science Prince of Songkla University Hat Yai Songkhla Thailand; ^2^ Department of Biology, Faculty of Science Mahasarakham University Kantharawichai Mahasarakham Thailand; ^3^ Department of Biology, Faculty of Science and Digital Innovation Thaksin University Papayom Phatthalung Thailand

**Keywords:** biogeography, Diptera, mitochondrial genome, phylogenetics, phylogeny

## Abstract

Species radiation in the family Drosophilidae has led to a diversity of species occupying a wide range of ecological niches. Despite the high diversity within this family, with over thousands of species and more than a hundred species recorded in Thailand and the Malay Peninsula, taxonomic classifications remain complicated due to morphological plasticity and inconsistent phylogenetic reconstructions based on limited genetic data. In this study, we assemble new mitochondrial genomes from *Drosophila* and *Scaptodrosophila* species collected in Thailand, expanding the genomic resources for these underexplored tropical regions. Phylogenetic analyses of 13 mitochondrial protein‐coding genes revealed well‐supported evolutionary relationships, with *Scaptodrosophila* forming a distinct lineage and several *Drosophila* subgroups, such as *ananassae*, *montium*, and *melanogaster*, exhibiting monophyly. Notable discrepancies were observed in the placement of the *suzukii* subgroup, which was not recovered as monophyletic, and the position of the *punjabiensis* subgroup, reflecting the complexities of lineage sorting and hybridization events. This comprehensive genomic analysis provides a more accurate understanding of evolutionary relationships within the family Drosophilidae's diversification in the tropics.

## Graphical abstract

The study expands genomic resources for *Drosophila* and *Scaptodrosophila* species by using mitochondrial genomes. Phylogenetic analyses revealed well‐supported evolutionary relationships, with *Scaptodrosophila* forming a distinct lineage and several *Drosophila* subgroups exhibiting monophyly, though discrepancies were found in the placement of the *suzukii* and *punjabiensis* subgroups.

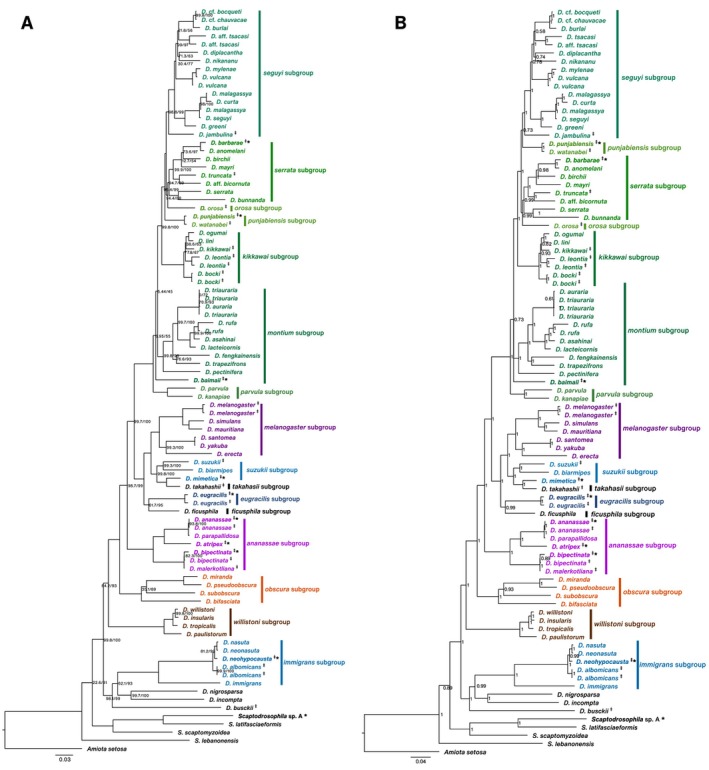

## 1 Introduction

Species diversification is a key process that increases biodiversity as lineages evolve to occupy different ecological niches through mechanisms such as mutation, epigenetic changes, genetic drift, and speciation (Naciri and Linder 2020). Biotic and abiotic factors, including climate, resource competition, predation, habitat persistence, and fitness trade‐offs, drive the diversification of insect species (Nosil and Crespi 2006; Peris and Condamine 2024; Spence and Tingley 2020). Such diversification often involves complex processes, including shifts in life‐history traits, dietary specialization, and habitat preferences, allowing insect species to colonize and persist in new niches over evolutionary timescales.

The family Drosophilidae consists of over a 1000 species worldwide (Brake and Bächli 2008). These species exhibit remarkable ecological diversity, ranging from opportunistic feeders on decaying fruits to specialists adapted to sap fluxes, flowers, and mushrooms (Markow and O'Grady 2008). In addition to temperate habitats, drosophilids occupy high‐altitude environments, desert conditions, and rainforest canopies in tropical regions, exhibiting ecological diversity and inhabiting various geographical regions (Izumitani et al. 2016). High reproductive rates, short generation times, broad range of ecological niches, and rapid adaptability have facilitated its member species to expand beyond their ancestral habitats (Markow and O'Grady 2008). Understanding the phylogenetic relationships among drosophilid species is important for understanding the mechanisms underlying this diversification.

Morphological plasticity and limitations in character selection create problems in Drosophilidae taxonomy (Moczek 2010), putting many species into species complexes. Characters traditionally used to differentiate species, such as wing venation patterns, body coloration, or bristle arrangement, can exhibit substantial intraspecific variation and overlapping phenotypes among closely related species (Coyne et al. 2004). In many cases, especially among cryptic or morphologically similar taxa, distinguishing between species requires detailed examination of male genitalia or other minute traits, which may be subject to developmental plasticity. As a result, some species groups have remained in a state of taxonomic uncertainty or have been put under species complexes, complicating biodiversity assessments (Jeffroy et al. 2006; Markow and O'Grady 2008).

Phylogenomic approaches using extensive genomic data have proven effective in resolving complex species relationships by providing higher resolution and revealing genome conservation across species (Chakraborty et al. 2021; Fontaine et al. 2015; Miller et al. 2017; Rane et al. 2019). Large‐scale genomic data allow for the recovery of thousands of orthologous loci and increase resolution and confidence in phylogenetic trees. These methods have been useful for studying groups that have experienced recent or rapid radiations, where shared polymorphisms and incomplete lineage sorting often confuse evolutionary relationships when only one or a few genes are analyzed (Jeffroy et al. 2006).

A classic example of such challenges is the *Drosophila nasuta* species complex of the *immigrans* group. This complex comprises around 12 morphologically similar species distributed across Southeast Asia (e.g., *Drosophila nasuta* Lamb, *Drosophila albomicans* (Duda), and *Drosophila kohkoa* Wheeler). The females of this complex are morphologically similar, whereas males can be distinguished by markings on the frons (Kitagawa et al. 1982). By examining genetic variation across several genetic loci, studies have provided a better understanding of species divergence and speciation processes within this complex (Bachtrog 2006; Mai et al. 2020; Yu et al. 1999).

The phylogeny of *Drosophila* has been extensively studied using both mitochondrial and nuclear markers (Conner et al. 2021; DeSalle et al. 2024; Yassin 2018; Yassin et al. 2016). Mitochondrial DNA (mtDNA) is a popular marker for phylogenetic analysis due to its high mutation rate, maternal inheritance, and low recombination events (Ballard and Whitlock 2004; Birky 1978; Brown et al. 1979). Thus, it is a powerful marker for species identification and resolving evolutionary history. Recently, DeSalle et al. (2024) performed a phylogenetic analysis of Drosophilidae using mitochondrial genomes, producing a well‐supported phylogeny. Similarly, Yassin et al. (2016) have emphasized the utility of combined mitochondrial and nuclear markers for disentangling the phylogenetic relationships among closely related *Drosophila* species, particularly in clades where rapid radiation has obscured evolutionary signals.

Despite extensive studies, significant gaps remain in our understanding of *Drosophila* diversity, especially in the tropical regions known for high species richness and endemism (Katoh et al. 2024). These gaps include the limited molecular data available for many species, the incomplete resolution of phylogenetic relationships, and the underrepresentation of some lineages in tropical ecosystems. The Malay Peninsula is home to several Dipteran species that are undescribed or poorly described regarding both taxonomy and evolutionary history (Detcharoen and Nilsai 2023; Grootaert 2009). This limited representation negatively impacts the greater understanding of how tropical ecosystems develop and maintain drosophilid biodiversity.

This study aimed to address this knowledge gap by using a phylogenomic approach to reconstruct the evolutionary history of *Drosophila* and *Scaptodrosophila* species from the Thai–Malay Peninsula tropical forests. We have assembled 35 additional mitochondrial genomes from raw reads obtained from the NCBI SRA, primarily from Conner et al. (2021) and Bronski et al. (2020). By using mitochondrial data, we aimed to better understand evolutionary relationships among these species and contribute to broader discussions on drosophilid classification and evolution. Our findings offer valuable information into the diversification of Drosophilidae in the tropics and help clarify their complex classifications.

## 2 Methods

### 2.1 Fly Collection and DNA Extraction

Nine *Drosophila* and one *Scaptodrosophila* species were collected from various places in Thailand using traps baited with fruits (Kimura and Suwito 2012) (Table 1). The collected flies were immediately put in 96% ethanol and were stored at −80°C upon arrival at the laboratory. The flies were classified morphologically following Markow and O'Grady (2006) under a stereo microscope. Genomic DNA was extracted from the whole individual fly using the phenol–chloroform method (Casaril et al. 2017). The quality and quantity of the extracted DNA were measured using agarose gel electrophoresis and Qubit4 with the Qubit dsDNA HS assay kit (Thermo Fisher Scientific, USA). The identification of the fly was supported using PCR with primers LCO1490 and HCO2198 (Folmer et al. 1994) targeting mitochondrial cytochrome c oxidase subunit I (COI) gene. The PCR amplicons were cleaned using the Exo‐CIP Rapid PCR Cleanup Kit (New England Biolabs, USA) and sent for Sanger sequencing (ATGC, Pathum Thani, Thailand). The sequences obtained were checked and blasted to NCBI GenBank to support species identification.

**TABLE 1:** Mitochondrial genome assembly statistics, GenBank accession numbers, and collection coordinates of the nine *Drosophila* and one *Scaptodrosophila* species.

Species	Size (bp)	%GC	Coverage (x)	Accession number	Collection site
*Ananassae* subgroup					
*D. ananassae*	15,821	21.7	1086.26	PP958477	6°06′08.3′′ N 101°50′53.8′′ E
*D. atripex*	15,805	21.5	2653.23	PP958478	13°43′55.0′′ N 100°32′19.8′′ E
*D. bipectinata*	15,778	21.2	178.49	PP958481	17°16′56.9′′ N 100°38′25.0″ E
*Eugracilis* subgroup					
*D. eugracilis*	16,714	20.8	218.97	PP958482	17°16′56.9′′ N 100°38′25.0″ E
*Hypocausta* subgroup					
*D. neohypocausta*	16,025	22.8	2755.77	PP958484	15°37′37.7′′ N 101°03′03.5′′ E
*Montium* group					
*D. baimaii*	15,472	21.6	353.97	PP958479	18°48′47.9′′ N 98°56′45.4″ E
*D. barbarae*	16,811	20.7	353.46	PP958480	18°30′01.9′′ N 99°15′54.7′′ E
*D. punjabiensis*	16,899	20.3	171.51	PP958485	18°30′01.9′′ N 99°15′54.7′′ E
*Suzukii* subgroup					
*D. mimetica*	16,770	20.5	903.83	PP958483	17°16′56.9′′ N 100°38′25.0′′ E
*Scaptodrosophila*					
*Scaptodrosophila* sp. A	15,664	21.3	301.33	PP958486	13°48′39.7′′ N 100°33′12.8′′ E

### 2.2 Nanopore Sequencing

The library preparation and sequencing steps were done according to the manufacturer's protocols. Briefly, about one microgram of DNA was repaired using the NEBNext Companion Module for Oxford Nanopore Technologies Ligation Sequencing (New England Biolabs, USA), cleaned using AMPure XP magnetic beads (Beckman Coulter, USA), and quantified for quantity with Qubit4 fluorometer (Thermo Fisher Scientific, USA). The cleaned DNA was ligated to barcodes and adaptors using the Native Barcoding Kit 24V14 (Oxford Nanopore Technologies, UK) and cleaned up again using the AMPure XP magnetic beads. The pooled library consisting of four to six barcoded samples was sequenced using a Nanopore MinION device with R10.4.1 flow cells. Each sequencing run was performed for about 48 h.

### 2.3 Assemblies

Reads were base‐called and demultiplexed using Dorado v0.6.0 (Oxford Nanopore Technologies, UK) with the high accuracy model. Fastp v0.23.4 (Chen 2023) with default parameters, was used to filter the reads. To *de novo* assemble the mitochondrial genome, amino acid sequences of the *Drosophila melanogaster* Meigen (GenBank accession number KT174474.1), *Drosophila ananassae* Doleschall (MK659805.1), *D. nasuta* (MK659831.1), and *Drosophila simulans* Sturtevant (MN046104.1) mitochondrial genes were used to construct a database to search for putative mitochondrial sequences of each fly species using Diamond v2.1.9.163 (Buchfink et al. 2021) with the fast sensitivity option. Those reads that matched were assembled using Flye v2.9.3‐b1797 (Kolmogorov et al. 2019) with default parameters. Bandage v0.8.1 (Wick et al. 2015) was used to visualize the assembly. The assembly was polished twice using pilon v1.24 (Walker et al. 2014). The sequencing depth of each mitochondrial genome was calculated by mapping the reads to the polished mitochondrial genome using minimap2 v2.27‐r1193 (Li 2018). The resulting file was used to compute the depth using the depth function in Samtools v1.18 (Danecek et al. 2021). In addition, 35 mitochondrial genomes from 33 species were assembled (Table A1). Illumina‐generated reads were downloaded from the NCBI SRA database, mainly from Conner et al. (2021) and Bronski et al. (2020). Unfortunately, we could not include the assemblies from DeSalle et al. (2024) as they are not publicly available at the time of the analysis. Quality control was performed using Fastp, where reads shorter than 50 bases were discarded. Putative mitochondrial sequences were identified using Diamond as described earlier. These reads were assembled using SPAdes v4.0.0 (Prjibelski et al. 2020) with k‐mers of 21, 33, 55, and 77. The resulting assembly graphs were visualized using Bandage.

### 2.4 Annotation

We used Mitos2 v2.1.9 (Donath et al. 2019) via Galaxy (Abueg et al. 2024) to annotate the mitochondrial genomes with RefSeq89 Metazoa as a reference. Gene annotations were rechecked manually by aligning them to the mitochondrial genes of *D. ananassae* . Additionally, the COI sequences amplified via PCR were checked by aligning them to the mitochondrial annotation to confirm their accuracy and consistency with the assembled mitochondrial genomes. For synteny analysis, the mitochondrial genomes were aligned using the progressive alignment function in Mauve v20150226 (Darling et al. 2004). The alignment backbone file was used as an input for visualization using the genoPlotR v0.8.11 package (Guy et al. 2011) in R v4.2.1 (R Core Team 2020).

### 2.5 Phylogenetic Analyses

We aligned each protein‐coding gene using Clustal Omega v1.1.0 (Sievers et al. 2011). ModelFinder (Kalyaanamoorthy et al. 2017) was used to search for the suitable substitution model for each gene. A maximum likelihood (ML) phylogenetic tree based on the concatenated 13 protein‐coding genes (10,324 bases) was constructed using IQ‐Tree v2.3.6 (Minh et al. 2020) with 10,000 ultrafast bootstraps and 10,000 SH approximate likelihood ratio tests. *Amiota setosa* Zhang & Chen (Diptera: Drosophilidae) was used as an outgroup. We also performed a Bayesian phylogenetic analysis of the same data using MrBayes v3.2.7a (Ronquist et al. 2012) with the GTR + Γ model with invariable sites. The analysis was run for 1,000,000 generations, with a sampling frequency of 1000, four chains, and a 25% relative burn‐in. Convergence was assessed by examining the trace files and diagnostics using Tracer v1.7.2 (Rambaut et al. 2018), including effective sample size values and trace plots. Trace plots were inspected to confirm stationarity and proper mixing of the chains, and the posterior distributions of the parameters were checked for consistency. All trees were visualized using FigTree v1.4.4 (Rambaut 2014).

### 2.6 Synonymous and Nonsynonymous Substitutions

We calculate synonymous (dS) and nonsynonymous (dN) substitutions of all protein‐coding genes in the mitochondrial genomes to infer their evolutionary rate of *ananassae*, *montium*, and *suzukii* subgroups (Table A2). The alignment was performed using Clustal Omega v1.1.0 (Sievers et al. 2011) and was used to calculate synonymous and nonsynonymous substitutions using the codeml function in PAML v4.9j (Yang 2007).

## 3 Results

We sequenced and analyzed mitochondrial genomes from nine *Drosophila* species and one *Scaptodrosophila* species collected from tropical areas in Thailand. Among these, mitochondrial genomes of six species including *Drosophila atripex* Bock & Wheeler, *D. baimaii* Bock & Wheeler, *D. barbarae* Bock & Wheeler, *D. punjabiensis* Parshad & Paika, and *D. mimetica* Bock & Wheeler, and *Scaptodrosophila* species A were reported for the first time. For *Scaptodrosophila* species A, species‐level identification could not be achieved, and its COI sequence did not match any known species in the NCBI database. The sequenced *Drosophila* mitochondrial genomes belonged to seven recognized groups: *D. barbarae* (*serrata* subgroup), *D. punjabiensis* (*punjabiensis* subgroup), *D. baimaii* (*montium* subgroup), *D. mimetica* (*suzukii* subgroup), *D. eugracilis* (*eugracilis* subgroup), *D. ananassae* , *D. atripex*, and *D. bipectinata* (*ananassae* subgroup), and *D. neohypocausta* (*immigrans* subgroup). Using Nanopore sequencing, the total number of reads generated per species ranged from 362,523 reads ( *D. barbarae* ) to 5,454,362 reads ( *D. mimetica* ), with approximately 86%–90% passing the quality control.

**FIGURE 1:**
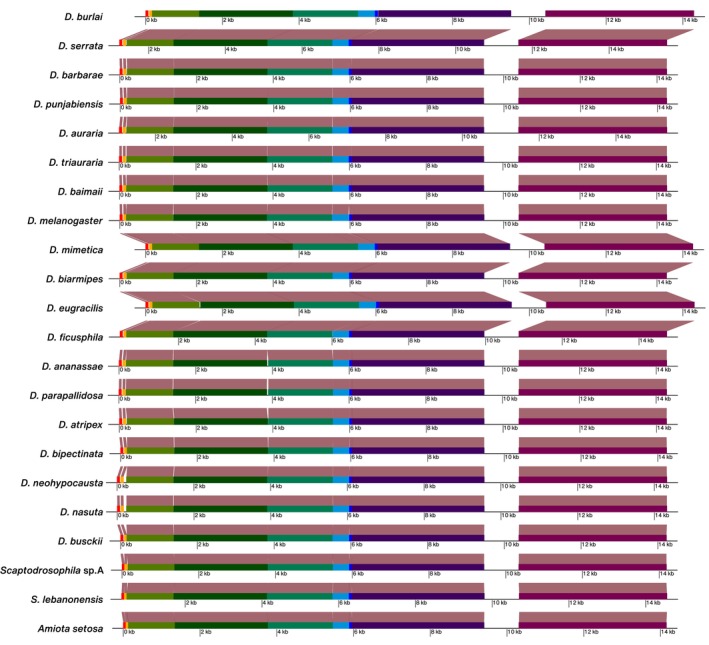
Genome synteny across *Drosophila* and *Scaptodrosophila* species. Each horizontal bar represents the genomic structure of a species, with syntenic blocks color‐coded to indicate conserved sequences. The scale bar provides a reference for genomic distances.

The mitochondrial genomes of the *ananassae* subgroup were around 15,800 bp with a GC content of 21.2%–21.7%. In contrast, the *eugracilis* subgroup exhibited a larger mitochondrial genome of 16,714 bp. The *montium* group species displayed mitochondrial genome sizes ranging from 15,472 to 16,899 bp. *Scaptodrosophila* sp. A had a mitochondrial genome size of 15,664 bp (Table 1). The synteny analysis revealed high evolutionary stability in the mitochondrial genomes across these species (Figure 1).

**FIGURE 2:**
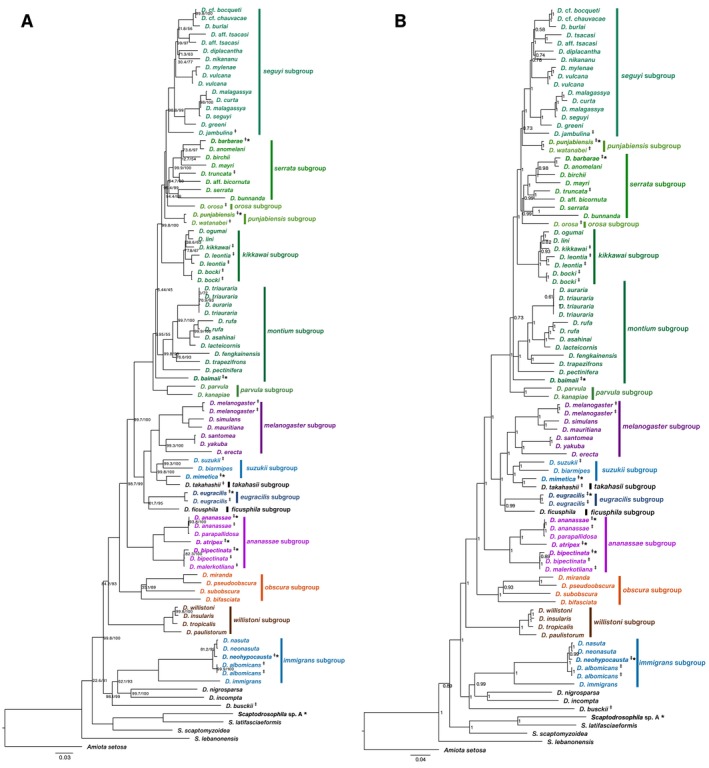
Phylogenetic trees of *Drosophila* and *Scaptodrosophila*. (A) Maximum likelihood phylogenetic tree constructed using 13 protein‐coding genes from mitochondrial genomes. The numbers at each node indicate ultrafast bootstrap and SH approximate likelihood ratio test values, respectively. Nodes with values of 100/100 are not shown for simplicity. (B) Bayesian inference phylogenetic tree constructed using the same gene dataset. Posterior likelihood values are shown at each node. The *Drosophila montium* group is highlighted in green colors. *Amiota setasa* was used as an outgroup.

Phylogenetic analyses of the concatenated 13 mitochondrial protein‐coding genes using both ML and Bayesian inference methods revealed overall congruent topologies for major *Drosophila* lineages (Figure 2). Throughout these analyses, *Scaptodrosophila* species consistently emerged as a distinct lineage apart from the main *Drosophila* clade. Within this lineage, *Scaptodrosophila* sp. A is closely related to *Scaptodrosophila latifasciaeformis*. Within *Drosophila*, the *seguyi*, *kikkawai*, *melanogaster*, *obscura*, and *ananassae* subgroups were consistently recovered as monophyletic in both ML and Bayesian trees.

Within the *ananassae* subgroup, *D. ananassae* , *D. atripex*, and *D. bipectinata* formed a well‐supported monophyletic group. Similarly, *D. neohypocausta* of the *hypocausta* subgroup was recovered with robust support as part of its respective lineage. Despite the broad congruence, the *suzukii* subgroup did not form a single clade in all analyses, with *D. mimetica* placed instead within the *takahashii* subgroup in both ML and Bayesian trees.

Within the monophyletic *montium* group, most species were recovered consistently between ML and Bayesian trees. However, the *punjabiensis* subgroup exhibited a notable discrepancy. Under ML, it emerged as sister to *orosa*, *serrata*, and *seguyi* subgroups, whereas in the Bayesian inference, it was placed at a more basal position in the *montium* clade. Furthermore, the placement of *D. baimaii* was inconsistent. It was not fully resolved in the Bayesian inference and had very low node support values in the ML analysis.

For mitochondrial genes in fly species from the *ananassae*, *montium*, and *suzukii* subgroups, the dN/dS ratios were less than one, indicating purifying selection (Table A2). Genes such as *ATP8*, *COX1*, and various NADH dehydrogenase components exhibited uniformly low dN/dS ratios across subgroups, supporting strong purifying selection.

## 4 Discussion

We sequenced the mitochondrial genomes of 10 tropical species, nine *Drosophila* and one *Scaptodrosophila*. The *de novo* assembly of these genomes showed that their sizes are consistent with other Drosophilidae, ranging from 15 to 16 kb (Karageorgiou et al. 2019; Wolff et al. 2016; Zhang and Jin 2020). Synteny analyses suggest evolutionary stability across the mitochondrial genomes among drosophilids. This conservation is consistent with previous reports in species including *Scaptodrosophila* (GenBank MK659851.1), *Drosophila incompta* Wheeler & Takada (De Ré et al. 2014), and *Drosophila subobscura* Collin (Karageorgiou et al. 2019).

Our analysis of the dN/dS ratio for mitochondrial genes across species from the *ananassae*, *montium*, and *suzukii* subgroups revealed purifying selection. Several NADH dehydrogenase genes exhibit low ratios across the species. This conservation of mitochondrial genes in *Drosophila* is maintained by purifying selection to retain efficient cellular respiration and energy production. Our findings align well with studies in other taxa, where purifying selection is a dominant force preserving the functionality of energy metabolism pathways (Baião et al. 2023; Cooper et al. 2015; Montooth et al. 2009).

### 4.1 Phylogenetic Analysis

Both ML and Bayesian approaches recovered broadly similar topologies, consistently placing *Scaptodrosophila* as a well‐supported outgroup to *Drosophila*. This corroborates previous nuclear and mitochondrial studies (Conner et al. 2021; DeSalle et al. 2024), confirming the evolutionary divergence between these genera. Within *Drosophila*, major clades such as the *melanogaster*, *obscura*, *ananassae*, and *kikkawai* subgroups emerged as monophyletic with high support. The *ananassae* subgroup was positioned as basal to the remaining *melanogaster* subgroups (DeSalle et al. 2024; Finet et al. 2021). However, the *suzukii* group was not recovered as monophyletic in either the ML or Bayesian analyses, potentially reflecting historical hybridization events or convergent mitochondrial patterns, similar to the previous study using the *Amyrel* gene (Da Lage et al. 2007).

The *Drosophila montium* species group, comprising subgroups such as *parvula*, *montium*, *kikkawai*, and *serrata*, is predominantly distributed across Asia and exhibits rapid lineage diversification (Yassin 2018). Our phylogenetic analysis indicates that the *parvula* subgroup diverged first within the *montium* group, similar to the study of Yassin (2018). However, the placement of *D. baimaii* remains contentious due to its very low bootstrap values, SH approximate likelihood ratio test, and posterior likelihood. Our results position it as a sister taxon to most *montium* members, reflecting the ambiguous status reported in nuclear gene analyses (Conner et al. 2021; Yassin 2018).

Discrepancies between our ML and Bayesian inference analyses further emphasize these challenges. For example, the position of the *punjabiensis* subgroup has relatively low node support values where the ML places it as sister to the *orosa*, *serrata*, and *seguyi* subgroups, whereas Bayesian inference suggests a closer relationship to the *seguyi* subgroup. This is also reported to be inconsistent by Conner et al. (2021), who suggested that *punjabiensis* is sister to *seguyi*, whereas Yassin et al. (2016) proposed it is sister to *serrata*, *kikawai*, and *seguyi*.

Similar to our findings, the analysis using 13 protein‐coding genes, rRNA, and tRNA by DeSalle et al. (2024) placed the *punjabiensis* subgroup as sister to the *seguyi* subgroup. Yet, *D. jambulina* was not placed within the *seguyi* subgroup but appeared in the same clade with other members of the *punjabiensis* subgroup. These inconsistencies may arise due to methodological differences or due to the limited resolution of mitochondrial data, suggesting the need for comprehensive genomic approaches to achieve a more accurate phylogenetic analysis. Moreover, incomplete lineage sorting, introgression, and differences in the evolutionary rates of mitochondrial and nuclear genomes may contribute to the observed differences within the *punjabiensis* subgroup (Ballard and Whitlock 2004; Toews and Brelsford 2012). Furthermore, mitochondrial genomes have a smaller effective population size, and recombination rates are low, which makes them more susceptible to genetic drift and faster lineage sorting than nuclear DNA (Pease and Hahn 2013; Toews and Brelsford 2012).

Despite these challenges, our analyses consistently support the monophyly of the *seguyi* subgroup. This finding aligns with previous studies and highlights the importance of dense taxon sampling in resolving fine‐scale phylogenetic relationships within the *montium* group (Conner et al. 2021; Yassin 2018). The observed diversity within this group likely reflects adaptations to varied ecological niches (Yassin 2018). Future studies should therefore incorporate nuclear genomic data and broader taxon sampling, which will be important in addressing the remaining ambiguities and providing further insight into the evolutionary dynamics of the *montium* species group.

The phylogeny of the family Drosophilidae has been challenging to resolve due to rapid radiation and incomplete lineage sorting (DeSalle et al. 2024). In our study, the mitochondrial phylogenetic trees placed several species into well‐supported clades, including a monophyletic *montium* species group. Our results also support previous studies confirming that all *montium* subgroups and *melanogaster* subgroups are sisters (Conner et al. 2021; DeSalle et al. 2024; Finet et al. 2021). Furthermore, our analyses separate the *Drosophila* and *Sophophora* subgenera, with the *Sophophora* clade showing recent divergence among species such as *Drosophila bipectinata* Duda, *D. melanogaster* , and *D. atripex*. For the subgenus *Drosophila*, our analyses strongly support *D. neohypocausta* as the sister species to *D. nasuta* .

*Scaptodrosophila* sp. A is closely related to *S. latifasciaeformis*, a species found in Africa and South America (Tidon 2006), and distantly from *S. lebanonensis*. Our findings support previous studies suggesting that the phylogenetic of *Scaptodrosophila* is more complex than previously thought (Russo et al. 2013; Van Der Linde et al. 2010; Yassin 2013). Factors such as incomplete lineage sorting, hybridization events, or uneven taxon sampling could drive its paraphyly. Molecular clock analyses estimate that *Scaptodrosophila* diverged from other drosophilids around 60 million years ago, concurrent with significant climatic shifts and the diversification of flowering plants in the late Paleocene to early Eocene (Russo et al. 1995; Wing et al. 2005). These environmental changes, including habitat preferences, resource usage, and mating strategies, may have provided ecological opportunities that allowed for *Scaptodrosophila* diversification.

The taxonomic status of *Scaptodrosophila* species A in our study remains uncertain because its COI sequence did not match any known species in the database. The failure to match in the database could mean that *Scaptodrosophila* species A is a new species. However, it is possible that this is due to missing data or low taxonomic sampling of the genus (Yassin 2013). Although *Scaptodrosophila* species A and *S. latifasciaeformis* suggest a close phylogenetic relationship, *Scaptodrosophila* species A might represent a distinct lineage. These findings together indicate that there is a poor understanding of the molecular and taxonomic knowledge of this genus and that there is a need to increase geographic and taxonomic sampling, especially in high biodiversity areas such as Southeast Asia.

### 4.2 Biogeographic and Evolutionary Implications

The ecological adaptations of the drosophilids studied here are diverse. Members of the *montium* group also show ecological diversity, which reflects ecological plasticity within the group. For example, *D. barbarae* and *D. punjabiensis* of the *serrata* and *punjabiensis* subgroups, respectively, are largely restricted to subtropical and forested habitats in Southeast Asia (Bock and Wheeler 1972). Similarly, *D. mimetica* (*suzukii* subgroup) is associated with ripening fruits and reflects its agricultural and ecological associations (Atallah et al. 2014; Hauser 2011). Species such as *D. ananassae* and *D. atripex* are generalists that feed on a broad diet and can be found throughout the year (Kimura and Suwito 2012). In contrast, *D. eugracilis* is frequently found in rainy seasons and in forests (Kimura and Suwito 2012).

Differences in ecological niches and behavioral traits (e.g., host fruit preferences, courtship rituals, and tolerance to temperature and humidity) drive the diversification of drosophilids (Markow and O'Grady 2006). The variety of habitats in Thailand, such as lowland rainforests, agricultural landscapes, and montane areas, provides multiple ecological slots for drosophilids to occupy. This mosaic of habitats promotes allopatric speciation and secondary contact zones where hybridization events can blur phylogenetic signals (Rundle and Nosil 2005; Via and West 2008). Thus, the ecology of these flies is inextricably linked to their evolutionary history, and further ecological studies may help pinpoint which traits have facilitated lineage divergence and adaptation.

## 5 Conclusion

In this study, we assembled and analyzed the mitochondrial genomes of 10 tropical drosophilid species, including nine *Drosophila* and one *Scaptodrosophila*. Including these tropical species in mitochondrial phylogenetic analysis is important for several reasons. As tropical regions contain a substantial share of global biodiversity, including tropical species ensures that the phylogenetic analysis reflects a broader range of genetic diversity. Our phylogenetic analyses confirmed the close evolutionary relationships among the *Drosophila* species and the distinct position of *Scaptodrosophila* within the Drosophilidae family. Furthermore, our study focuses on species collected from specific tropical regions. Expanding the genomic dataset will further refine phylogenetic relationships and contribute to a better understanding of drosophilid evolution and taxonomy.

## Author Contributions

**Matsapume Detcharoen:** conceptualization (equal), formal analysis (equal), funding acquisition (equal), investigation (equal), methodology (equal), resources (equal), software (equal), validation (equal), writing – original draft (equal), writing – review and editing (equal). **Pairot Pramual:** funding acquisition (equal), resources (equal), supervision (equal), writing – review and editing (equal). **Areeruk Nilsai:** investigation (equal), resources (equal), writing – review and editing (equal).

## Conflicts of Interest

The authors declare no conflicts of interest.

## Data Availability

Mitochondrial genomes were deposited on NCBI GenBank with accession numbers PP958477–PP958486. Raw reads were deposited on SRA with the project number PRJNA1158853.

## Appendix A

**TABLE A1:** List of additional mitochondrial genomes used for phylogenetic analysis.

Species	Sequencing run/GenBank accession number
*Amiota setosa*	OP381036.1
*Drosophila* aff.*bicornuta*	SRR13188910
*Drosophila* aff.*tsacasi*	SRR13188914
*Drosophila albomicans*	KT119344.1
*Drosophila albomicans*	NC027937.1
*Drosophila ananassae*	MK659805.1
*Drosophila anomelani*	SRR13188938
*Drosophila asahinai*	SRR9997934
*Drosophila auraria*	MK659808.1
*Drosophila biarmipes*	MK659809.1
*Drosophila bifasciata*	MK659810.1
*Drosophila bipectinata*	MK659811.1
*Drosophila birchii*	SRR9997935
*Drosophila bocki*	SRR9997933
*Drosophila bocki*	SRR13188909
*Drosophila bunnanda*	SRR9997939
*Drosophila burlai*	MK742872.1
*Drosophila busckii*	MT429169.1
*Drosophila* cf.*bocqueti*	SRR13188908
*Drosophila* cf.*chauvacae*	SRR13188935
*Drosophila curta*	MK742874.1
*Drosophila diplacantha*	SRR13188934
*Drosophila erecta*	MK659815.1
*Drosophila eugracilis*	MK659816.1
*Drosophila fengkainensis*	SRR13188933
*Drosophila ficusphila*	MK659817.1
*Drosophila greeni*	SRR13188932
*Drosophila immigrans*	OY757235.1
*Drosophila incompta*	KM275233.1
*Drosophila insularis*	OP208750.1
*Drosophila jambulina*	SRR9997925
*Drosophila kanapiae*	SRR9997931
*Drosophila kikkawai*	MK659822.1
*Drosophila lacteicornis*	SRR9997940
*Drosophila leontia*	SRR9997936
*Drosophila leontia*	MK742877.1
*Drosophila lini*	SRR13188931
*Drosophila malagassya*	SRR13188930
*Drosophila malagassya*	MK742878.1
*Drosophila malerkotliana*	MK659826.1
*Drosophila mauritiana*	AF200830.1
*Drosophila mayri*	SRR9997930
*Drosophila melanogaster*	KP843851.1
*Drosophila melanogaster*	KJ947872.2
*Drosophila miranda*	MK659828.1
*Drosophila mylenae*	MK742879.1
*Drosophila nasuta*	MK659831.1
*Drosophila neonasuta*	MK659833.1
*Drosophila nigrosparsa*	MK659834.1
*Drosophila nikananu*	SRR9997927
*Drosophila ogumai*	SRR13188929
*Drosophila orosa*	SRR13188927
*Drosophila parapallidosa*	MK659836.1
*Drosophila parvula*	SRR13188925
*Drosophila paulistorum*	OP208741.1
*Drosophila pectinifera*	SRR9997937
*Drosophila pseudoobscura*	FJ899745.1
*Drosophila rufa*	SRR9997942
*Drosophila rufa*	MK742881.1
*Drosophila santomea*	KF824871.1
*Drosophila seguyi*	SRR9997926
*Drosophila serrata*	MK659841.1
*Drosophila simulans*	CM016414.1
*Drosophila subobscura*	MG421017.1
*Drosophila suzukii*	KU588141.1
*Drosophila takahashii*	MK659844.1
*Drosophila trapezifrons*	SRR13188924
*Drosophila triauraria*	SRR9997941
*Drosophila triauraria*	SRR13188919
*Drosophila triauraria*	MK659846.1
*Drosophila tropicalis*	OP208759.1
*Drosophila truncata*	SRR9997922
*Drosophila tsacasi*	MK742883.1
*Drosophila vulcana*	SRR9997929
*Drosophila vulcana*	MK742886.1
*Drosophila watanabei*	SRR9997920
*Drosophila willistoni*	OP208742.1
*Drosophila yakuba*	KF824874.1
*Scaptodrosophila lebanonensis*	MK659851.1
*Scaptodrosophila latifasciaeformis*	SRR26246298
*Scaptodrosophila scaptomyzoidea*	SRR26246564

**TABLE A2:** Nonsynonymous to synonymous substitutions (dN/dS) ratio of *Drosophila* species of *ananassae*, *montium*, and *suzukii* subgroups.

	dN	dS	dN/dS
*Ananassae* subgroup ( *D. ananassae* , *D. atripex*, *D. bipectinata* , *D. malerkotliana*, and *D. parapallidosa*)			
atp6	0.0069	1.7780	0.003881
atp8	0.0001	0.8921	0.000112
cob	0.0146	2.1153	0.006902
cox1	0.0023	2.2024	0.001044
cox2	0.0049	1.3034	0.003759
cox3	0.0073	1.7655	0.004135
nad1	0.0062	3.8193	0.001623
nad2	0.0257	1.7356	0.014808
nad3	0.0227	5.0310	0.004512
nad4	0.0138	4.4538	0.003098
nad4l	0.0001	0.0086	0.011628
nad5	0.0174	2.9082	0.005983
nad6	0.0334	2.4529	0.013617
*Montium* group (*D. baimaii*, *D. barbarae* , *D. punjabiensis*, *D. auraria*, and *D. kikawai*)			
atp6	0.0261	5.9274	0.004403
atp8	0.0377	3.5518	0.010614
cob	0.0204	6.6552	0.003065
cox1	0.0045	5.5705	0.000808
cox2	0.0164	5.0864	0.003224
cox3	0.0337	4.5484	0.007409
nad1	0.0294	8.5904	0.003422
nad2	0.0963	4.8960	0.019669
nad3	0.0669	13.409	0.004989
nad4	0.0683	8.9105	0.007665
nad4l	0.0229	3.4987	0.006545
nad5	0.0800	10.1852	0.007855
nad6	0.1246	9.6580	0.012901
*Suzukii* subgroup ( *D. mimetica* , *D. biarmipes*, and *D. suzukii*)			
atp6	0.0036	1.5239	0.002362
atp8	0.0002	1.9016	0.000105
cob	0.0051	1.435	0.003554
cox1	0.0015	1.5675	0.000957
cox2	0.0067	1.4563	0.004601
cox3	0.0090	1.5052	0.005979
nad1	0.0050	3.3761	0.001481
nad2	0.0096	1.2423	0.007728
nad3	0.0103	3.2886	0.003132
nad4	0.0072	3.1973	0.002252
nad4l	0	0.4557	0.0001
nad5	0.0157	6.6326	0.002367
nad6	0.0314	3.8322	0.008194
